# Patient preferences for scar orientation after gender-affirming mastectomy: A survey study

**DOI:** 10.1016/j.jpra.2026.06.011

**Published:** 2026-07-01

**Authors:** Lindsay A. Tao, Carolyn Cafro, Esther A. Kim

**Affiliations:** a University of California, San Francisco, Department of Surgery, Division of Plastic Surgery, San Francisco, CA, USA; b University of Rochester School of Medicine and Dentistry, Rochester, NY, USA

**Keywords:** Gender-affirming mastectomy, Transgender surgery, Scar orientation, Incision pattern, Aesthetic outcomes, Patient preferences, Top surgery

## Abstract

**Background:**

Gender-affirming mastectomy (GAM) most commonly uses the double-incision technique, producing prominent transverse chest scars. Although effective, these scars are highly visible and may influence postoperative aesthetic satisfaction. Incision orientation is typically determined by the surgeon, yet little is known about which patterns patients find favorable. This study evaluated patient preferences for double incision orientations to inform preoperative discussions.

**Methods:**

A cross-sectional survey was distributed to individuals with a history of or considering GAM. Participants ranked connected and non-connected incision patterns from most to least aesthetically pleasing. Rankings were converted to weighted preference scores using the Borda count method. The Friedman test, with post hoc Wilcoxon signed-rank tests and Bonferroni correction, assessed preference differences.

**Results:**

104 responses were analyzed. Participants were primarily 20–29 (49%) or 30–39 years old (28%), identified as transgender men (66%) or non-binary (31%), and mostly underwent GAM (85%). Friedman testing demonstrated significant differences among incision types (*p* < 0.001). Curved incisions were most strongly preferred across connected and non-connected designs (all *p* < 0.001). In non-connected designs, curved incisions received the highest weighted score (460), ranked first by 66% of participants, followed by angular incisions (345). In connected designs, curved incisions again ranked highest (451; 65% first-choice), followed by inverted V incisions (298). Large V and inverse-diagonal patterns were least favored.

**Conclusions:**

Patients considering double-incision GAM show varied scar orientation preferences, with curved incisions following the natural pectoralis contour most favored. Discussing scar orientation may support more informed preoperative discussions and shared decision-making when multiple surgical options are feasible.

## Introduction

Gender-affirming surgery (GAS) plays a critical role in the care of transgender and gender diverse individuals experiencing gender dysphoria. Chest masculinization surgery, or gender-affirming mastectomy (GAM), is among the most commonly performed gender-affirming procedures and has been associated with high levels of patient satisfaction, with reported satisfaction rates exceeding 90% following surgery.^1^ Current standards of care recognize GAS as a medically necessary treatment for many patients and emphasize its importance in aligning physical characteristics with gender identity.^2^ As access to gender-affirming care has expanded, the demand for these procedures has increased substantially in recent decades.^3^

Multiple surgical techniques have been described for GAM, with incision placement and scar orientation varying depending on patient anatomy, breast size, and skin elasticity. Common approaches include periareolar and double-incision mastectomy with or without free nipple grafting, each producing distinct postoperative scar patterns across the chest.^4^^,^^5^ Prior studies evaluating outcomes after GAM have largely focused on surgical safety, complication rates, aesthetic outcomes, and overall patient satisfaction. While these metrics provide important insight into the effectiveness of operative techniques, relatively little attention has been focused directly on patient preferences regarding the orientation and appearance of postoperative scars. Prior investigations have examined public perceptions of chest masculinization outcomes, including preferences regarding scar contour and nipple-areolar complex characteristics.^6^ However, these studies primarily evaluated observer assessments of masculinity rather than the preferences of transgender and gender-diverse individuals themselves. As a result, little is known about how patients seeking or undergoing GAM perceive different scar orientations.

Scar placement is an inherent component of most GAM techniques and represents a visible and permanent aspect of surgical outcomes. Although incision selection is typically guided by anatomical considerations, patient preferences regarding scar orientation may also influence postoperative satisfaction and perceived aesthetic outcomes. In particular, among patients who are candidates for double-incision mastectomy, there is often flexibility in the design and orientation of the resulting transverse scars. These incisions may be configured in a variety of orientations, including curved, straight, or angular patterns across the chest. Because scar orientation is most variable within this approach, the present study focuses specifically on scar patterns generated by the double-incision technique.

To date, no studies have specifically evaluated whether transgender men or non-binary individuals have preferences regarding these variations in scar orientation within the double-incision approach. The purpose of the present study was to evaluate patient preferences for scar orientation following double-incision GAM and to assess how these preferences may inform preoperative counseling and shared decision-making. Of note, aesthetic preference represents a distinct construct from postoperative satisfaction, patient-reported outcomes, and clinical effectiveness; accordingly, this study was designed to characterize patient perspectives regarding scar orientation rather than evaluate the comparative outcomes of different incision designs.

## Methods

### Study design

This cross-sectional survey study was approved by the University of California, San Francisco Institutional Review Board. Eligible participants included adults identifying as transgender men or non-binary individuals who had previously undergone or were considering GAM. Participants were identified through clinical records and contacted via institutional patient email lists. All participants were current or former patients of the senior author. Participation was voluntary and uncompensated.

### Survey instrument

The survey was designed by the authors based on clinical experience with GAM and prior observations of patient preferences regarding incision orientation. To evaluate aesthetic preferences, twelve schematic illustrations were created to represent realistic surgical options within the double-incision approach based on chest wall contour and skin redundancy. These consisted of six connected incision types (U-shaped, straight, upside-down V, large V, curved, and inverted V) and six non-connected incision types (U-shaped, straight, inverse-diagonal, diagonal, curved, and angular). Each illustration depicted a neutral, black-and-white outline of the torso with digitally rendered incision lines representing scar orientation. Schematic illustrations were intentionally selected to isolate scar orientation and facilitate standardized comparison across incision designs. This allowed controlled comparison of incision geometry while minimizing potential confounding visual variables such as skin tone, lighting, chest morphology, scar quality, contour irregularities, and postoperative healing characteristics. (Supplemental Table 1).

Participants ranked the incision types within each category (connected and non-connected) using a forced ranking scale from 1 (most aesthetically pleasing) to 6 (least aesthetically pleasing). Ties were not permitted. Additional survey items collected demographic and surgical data, including age, gender identity, race and ethnicity, history of prior top surgery, and type of prior mastectomy, if applicable. The survey was administered anonymously via Qualtrics (Qualtrics, Provo, UT, USA), and all responses were stored securely in de-identified form.

### Data collection

A total of 442 eligible patients were invited to participate via email. Of these, 118 individuals initiated the survey, and 104 completed all ranking questions and were included in the final analysis, yielding an overall response rate of 24% and a completion rate of 88% among respondents. Incomplete responses were excluded. Responses were collected over a six-week period.

### Statistical analysis

Incision preference rankings were converted into weighted scores using a Borda count method, assigning 5 points to a first-place ranking, 4 points to second place, and sequentially decreasing to 0 point for sixth place. With 104 respondents, weighted scores had a range of 0 to 520, with higher scores indicating greater overall preference.

Additional subgroup rankings by gender identity were calculated descriptively using the same Borda scoring method for transgender male (*n* = 69) and non-binary respondents (*n* = 32). Because subgroup sizes differed, the possible weighted score ranges were 0 to 345 and 0 to 160, respectively. Subgroup sample sizes reflect the number of respondents within each gender identity category who completed all ranking questions and were included in the final analysis. Three respondents identifying as “other” were excluded from this descriptive subgroup analysis. Subgroup rankings by history of GAM were calculated descriptively using the Borda scoring method for prior GAM (*n* = 88) and no history of GAM (*n* = 16). Possible weighted score ranges were 0 to 440 and 0 to 80, respectively. The aforementioned analyses were descriptive in nature and were not powered for formal statistical comparison.

The Friedman test was used to evaluate global differences in ordinal rankings across incision types within connected and non-connected groups. The alpha level was set a priori at 0.05 for global comparisons. Pairwise post hoc comparisons were performed using Wilcoxon signed-rank tests with Bonferroni correction for multiple comparisons. Given 15 pairwise comparisons per category, the Bonferroni-adjusted significance threshold was 0.0033.

Descriptive statistics were used to summarize demographic variables. All analyses were performed using Microsoft Excel (Microsoft Corp., Redmond, WA) and R version 4.1.1 (R Foundation for Statistical Computing, Vienna, Austria).

## Results

### Participant characteristics

Of 442 eligible patients invited to participate, 118 initiated the survey and 104 completed all ranking questions and were included in the final analysis. Participants were predominantly between 20 and 29 years of age (48.98%), followed by 30 to 39 years (27.55%). Most respondents identified as transgender men (66.3%), with 30.8% identifying as non-binary and 2.3% identifying as other. All participants were assigned female at birth. The majority had previously undergone GAM (84.62%), most commonly double incision with free nipple grafting (78.14%). The cohort was primarily White (68.37%), followed by Latinx (11.22%), Asian (10.2%), and mixed race (11.22%) individuals (Table 1).

**Table 1:** Patient demographics.

*Demographic*	*N (%)*
All Participants	104 (100)
Age (yr)	
18–19	7 (7.1)
20–29	48 (49)
30–39	27 (27.6)
40–49	11 (11.2)
50+	11 (11.2)
Gender	
Transgender Man	69 (66.3)
Non-binary	32 (30.8)
Other	3 (2.9)
Sex Assigned at birth	
Female	104 (100)
Prior GAM?	
Yes	88 (84.6)
No	16 (15.4)
Incisional Method of GAM	
Double Incision w/ FNG	78 (78.1)
Double Incision w/out FNG	7 (8)
Periareolar	3 (3.4)
Ethnicity	
White	67 (64.4)
Latinx	11 (10.6)
Asian	10 (9.6)
Mixed	11 (10.6)
Black	3 (2.9)
Middle Eastern	1 (1)
Other	1 (1)

GAM: Gender-affirming mastectomy; FNG: Free nipple graft.

### Non-Connected incision preferences

**Fig. 1:**
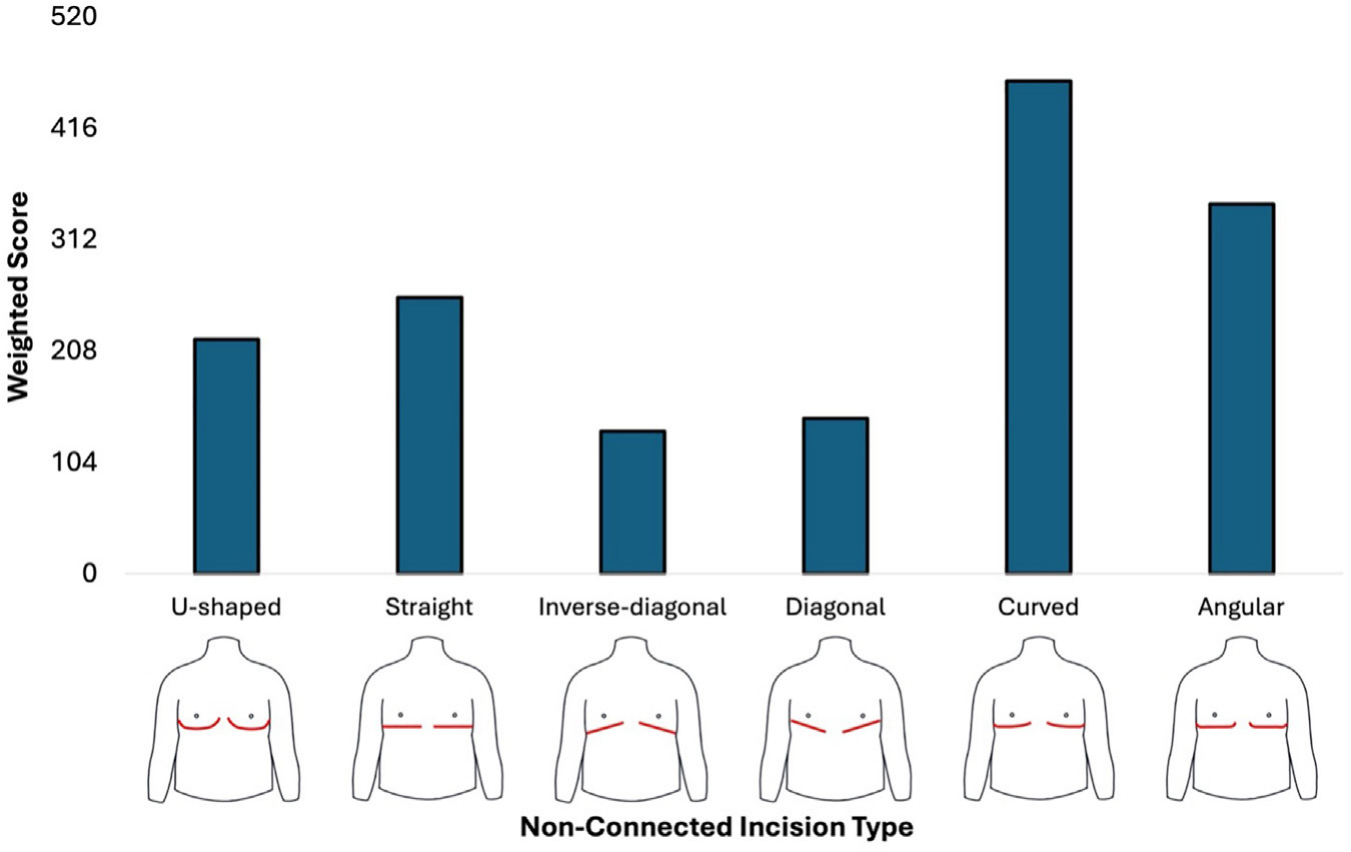
Weighted preference scores for non-connected incision types following gender-affirming mastectomy. Scores were calculated using the Borda count method based on participant rankings (*n* = 104), with higher scores indicating greater overall preference. Schematic illustrations depict the incision orientations evaluated in the survey.

Significant overall differences were observed among non-connected incision types on Friedman testing (*p* < 0.001). Weighted preference scores and rank distributions are summarized in Table 2 and illustrated in Fig. 1. Curved incisions received the highest weighted score (460) and were ranked first by 66.3% of participants, with only 1.9% ranking them last. Angular incisions demonstrated the second highest weighted score (345) and were most frequently ranked second (41.3%). Straight (258) and U-shaped (219) incisions occupied intermediate positions with more distributed ranking patterns. In contrast, inverse-diagonal (133) and diagonal (145) incisions received the lowest weighted scores and were most frequently ranked in the fifth or sixth positions.

**Table 2:** Non-connected GAM incision preferences.

	1 (best)	2	3	4	5	6 (worst)	Weighted Score
U-shaped	11 (10.6)	15 (14.4)	24 (23.1)	12 (11.5)	8 (7.7)	34 (32.7)	219
Straight	8 (7.7)	17 (16.3)	22 (21.2)	32 (30.8)	20 (19.2)	5 (4.8)	258
Inverse-diagonal	2 (1.9)	3 (2.9)	12 (11.5)	20 (19.2)	35 (33.7)	32 (30.8)	133
Diagonal	1 (1)	4 (3.8)	12 (11.5)	27 (26)	34 (32.7)	26 (25)	145
Curved	69 (66.3)	22 (21.2)	6 (5.8)	4 (3.8)	1 (1)	2 (1.9)	460
Angular	13 (12.5)	43 (41.3)	28 (26.9)	9 (8.7)	6 (5.8)	5 (4.8)	345

Data are presented as number of responses with percentage of total responses for each incision type. Weighted scores were calculated on a scale from 0 to 520, with higher scores indicating greater overall preference.

On pairwise Wilcoxon signed-rank testing with Bonferroni correction (α = 0.0033), curved incisions were ranked significantly higher than all other non-connected incision types (all adjusted *p* < 0.001). Angular and straight incisions were each preferred significantly over inverse-diagonal and diagonal designs. No significant difference was observed between U-shaped and straight incisions or between U-shaped and diagonal incisions after correction (Supplemental Table 1).

### Connected incision preferences

**Fig. 2:**
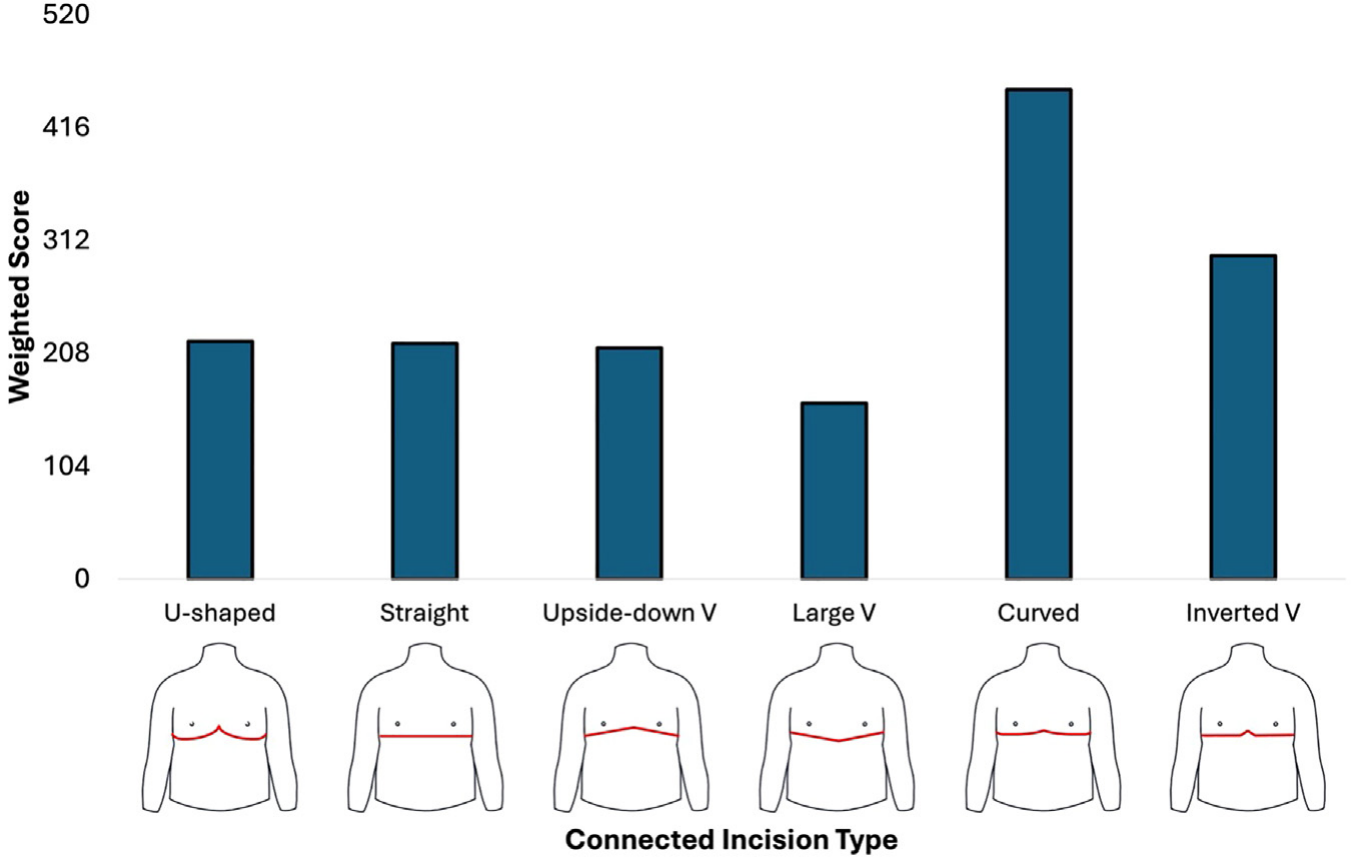
Weighted preference scores for connected incision types following gender-affirming mastectomy. Scores were calculated using the Borda count method based on participant rankings (*n* = 104), with higher scores indicating greater overall preference. Representative schematic illustrations of each incision orientation are shown.

Friedman testing similarly demonstrated significant overall differences among connected incision types (*p* < 0.001). Weighted scores and rank distributions are presented in Table 3 and illustrated in Fig. 2. Curved incisions again received the highest weighted score (451) and were ranked first by 65.4% of participants, with minimal last-place rankings, indicating strong consensus. Inverted V incisions demonstrated the second highest weighted score (298) and were most frequently ranked second (34.6%). U-shaped (219) and straight (217) incisions demonstrated similar intermediate rankings with broader distribution across positions. Large V incisions received the lowest weighted score (162) and were frequently ranked among the least preferred options.

**Table 3:** Connected GAM incision preferences.

	1 (best)	2	3	4	5	6 (worst)	Weighted Score
U-shaped	11 (10.6)	19 (18.3)	16 (15.4)	16 (15.4)	8 (7.7)	34 (32.7)	219
Upside-down V	3 (2.9)	13 (12.5)	25 (24)	20 (19.2)	31 (29.8)	12 (11.5)	213
Straight	7 (6.7)	13 (12.5)	19 (18.3)	23 (22.1)	27 (26)	15 (14.4)	217
Large V	5 (4.8)	2 (1.9)	17 (16.3)	25 (24)	28 (26.9)	27 (26)	162
Inverted V	10 (9.6)	36 (34.6)	20 (19.2)	18 (17.3)	8 (7.7)	12 (11.5)	298
Curved	68 (65.4)	21 (20.2)	7 (6.7)	2 (1.9)	2 (1.9)	4 (3.8)	451

Data are presented as number of responses with percentage of total responses for each incision type. Weighted scores were calculated on a scale from 0 to 520, with higher scores indicating greater overall preference.

Post hoc Wilcoxon testing with Bonferroni correction demonstrated that curved incisions were ranked significantly higher than all other connected designs (all adjusted *p* < 0.001). Large V incisions were ranked significantly lower than straight and inverted V designs, and upside-down V incisions were ranked significantly lower than inverted V designs. No significant differences were observed between U-shaped and upside-down V, straight, large V, or inverted V incisions after correction (Supplemental Table 2).

### Subgroup analyses

Overall ranking patterns were highly similar between transgender male and non-binary respondents (Table 4). Curved incisions remained the highest-ranked design across both groups for both non-connected and connected scar configurations. Minor differences were observed in the ordering of several lower and intermediate ranked designs. Among non-connected incisions, diagonal and inverse-diagonal orientations were reversed between groups, with transgender men ranking diagonal higher than inverse-diagonal (105 vs. 80) while non-binary respondents ranked inverse-diagonal higher than diagonal (47 vs. 35). Among connected incisions, straight and U-shaped designs differed in relative ordering, with transgender men ranking U-shaped higher than straight (161 vs. 132), whereas non-binary respondents ranked straight higher than U-shaped (79 vs. 56). Despite these small variations, the overall preference pattern remained comparable across gender identities.

**Table 4:** Incision preference rankings by gender identity and scar configuration.

	Non-Connected		Connected	
Weighted Rank Order	Transgender Male (*n* = 69)	Non-binary (*n* = 32)	Transgender Male (*n* = 69)	Non-binary (*n* = 32)
1	Curved (304)	Curved (141)	Curved (301)	Curved (135)
2	Angular (227)	Angular (110)	Inverted V (195)	Inverted V (93)
3	Straight (170)	Straight (84)	U-shaped (161)	Straight (79)
4	U-Shaped (149)	U-Shaped (63)	Upside-down V (142)	Upside-down V (65)
5	Diagonal (105)	Inverse-Diagonal (47)	Straight (132)	U-shaped (56)
6	Inverse-Diagonal (80)	Diagonal (35)	Large V (104)	Large V (52)

Subgroup sizes reflect respondents within each gender identity category who completed all ranking questions; three respondents identifying as “other” were excluded from subgroup analysis due to small sample size. Values represent weighted preference scores, with higher scores indicating greater overall preference. Weighted scores were calculated by assigning decreasing point values to ranked choices and summing responses across participants within each group. Rankings (1–6) reflect the order of preference based on total weighted score within each subgroup. Score ranges differed by subgroup due to sample size, with possible totals ranging from 0–345 for transgender male respondents and 0–160 for non-binary respondents. Bolded values indicate incision types with different rank positions between transgender male and non-binary respondents within the same scar configuration.

To assess the potential influence of prior surgical experience on incision preferences, a descriptive comparison was performed between respondents with prior GAM (*n* = 88) and those without prior GAM (*n* = 16). Preference patterns were highly consistent between groups (Table 5). Among non-connected incision designs, both cohorts demonstrated identical rank ordering, with curved incisions ranked highest, followed by angular, straight, and U-shaped designs. Similarly, among connected incision designs, curved and inverted V incisions were the highest-ranked designs in both groups. Minor differences were observed only in the relative ordering of straight and U-shaped incisions. No formal statistical comparisons were performed because of the limited size of the no-prior-GAM cohort.

**Table 5:** Incision preference rankings by history of GAM and scar configuration.

	Non-Connected		Connected	
Weighted Rank Order	Prior GAM (*n* = 88)	No Prior GAM (*n* = 16)	Prior GAM (*n* = 88)	No Prior GAM (*n* = 16)
1	Curved (391)	Curved (69)	Curved (383)	Curved (68)
2	Angular (295)	Angular (50)	Inverted V (252)	Inverted V (46)
3	Straight (214)	Straight (44)	U-shaped (187)	Straight (33)
4	U-Shaped (187)	U-Shaped (32)	Straight (184)	U-shaped (32)
5	Diagonal (120)	Diagonal (25)	Upside-down V (182)	Upside-down V (31)
6	Inverse-Diagonal (113)	Inverse-Diagonal (20)	Large V (132)	Large V (30)

Values represent weighted preference scores calculated using the Borda count method, with higher scores indicating greater overall preference. Participants ranked each incision design from 1 (most preferred) to 6 (least preferred); scores were assigned from 5 to 0 points, respectively, and summed across respondents within each subgroup. Rankings (1–6) reflect the order of preference based on total weighted score within each cohort. Respondents without prior GAM included both individuals considering future surgery and individuals without a history of or current plans for surgery. Comparisons are descriptive only. Score ranges differed by subgroup due to sample size, with possible totals ranging from 0–440 for prior-GAM respondents and 0–80 for no prior-GAM respondents. Bolded values indicate incision types with different rank positions between history of GAM and no history of GAM respondents within the same scar configuration.

## Discussion

This study evaluated patient preferences for scar orientation following double-incision GAM and found that preferences are heterogeneous across individuals, with curved incision patterns that follow the contour of the pectoralis border receiving the highest overall ratings. Although curved incisions demonstrated the strongest consensus preference, every incision pattern evaluated was selected as the most aesthetically favorable by at least one participant, underscoring the diversity of aesthetic goals among individuals seeking or considering GAM. These findings suggest that preferences regarding scar design are not uniform and should not be assumed at the individual patient level. Rather, they highlight the importance of eliciting individual patient preferences during preoperative discussions when multiple technically feasible scar configurations are available. Of import, the observed preference rankings reflect relative aesthetic preferences among survey respondents and should not be interpreted as evidence of superior clinical outcomes, postoperative satisfaction, or surgical effectiveness associated with any particular incision design.

Incisional approach in GAM is not necessarily an independently modifiable variable, but rather is constrained by patient anatomy, including breast size, skin redundancy, and nipple areolar complex (NAC) considerations, which together determine the range of feasible surgical approaches.^7^ The incision patterns evaluated in this study reflect aesthetic preference in a standardized, theoretical context specifically in the double-incision approach rather than a set of universally interchangeable surgical options. In clinical practice, these findings are most applicable to preoperative counseling and shared decision-making in scenarios when pursuing the double-incision technique.

Chest masculinization surgery has been shown to significantly improve gender dysphoria, body image, and overall quality of life for transgender and non-binary individuals.^8^^,^^9^ Prior work evaluating aesthetic outcomes after top surgery has emphasized the importance of chest contour, NAC placement, and scar characteristics in shaping overall patient satisfaction.^10^ Studies examining aesthetic perception of masculinized chests have similarly demonstrated that scars placed along the inferior border of the pectoralis muscle are frequently perceived as more masculine and aesthetically favorable.^6^ These studies hypothesize that scar orientations that mimic or accentuate the pectoralis contour may best approximate the appearance of a well-developed pectoralis muscle, a feature commonly associated with chest masculinity.^11^ The present findings build on this literature by demonstrating that patients themselves intuitively exhibit strong preferences for incision designs that follow natural chest wall contours, particularly curved orientations that mimic the pectoralis border.

Overall ranking patterns were broadly similar between transgender male and non-binary respondents. Curved incisions remained the highest-ranked design across both groups for both connected and non-connected scar configurations, and the relative ordering of most incision types was comparable. Although minor differences in rank ordering were observed, these subgroup analyses were descriptive only and no formal statistical comparisons were performed because of the limited sample size, particularly among non-binary respondents. Due to these limitations, findings should be interpreted cautiously and are best viewed as hypothesis-generating. Nevertheless, prior research suggests that transgender and non-binary individuals may pursue GAS with different aesthetic goals and priorities.^12^ While the present study was not powered to evaluate such differences, larger and more diverse cohorts are needed to determine whether meaningful differences in incision orientation preferences exist across gender identities.

Current standards of care emphasize that gender-affirming surgical care should be tailored to patient anatomy, identity, and aesthetic goals rather than a uniform operative template. In practice, surgical technique selection is guided by factors such as breast size, skin elasticity, and degree of ptosis.^9^ Patients with smaller chest size, minimal skin redundancy, and good skin elasticity may be candidates for periareolar or limited-incision techniques, whereas patients with larger breast size, greater ptosis, or reduced skin elasticity more often require double-incision mastectomy with or without free nipple grafting to achieve adequate contour and skin resection. Additionally, factors such as NAC position and chest wall contour influence incision placement and the ability to achieve a flat, tension-free closure. In patients with larger body habitus, connecting incisions across the midline may be necessary to maintain a smooth and contiguous chest contour. Representative examples of these techniques and corresponding scar patterns have been well described in prior literature, including periareolar, double-incision approaches with different scar orientations. These references provide helpful visual examples of how incision placement and scar orientation translate to clinical practice. While overall incision strategy is ultimately dictated by these anatomic factors, regardless of patient preference, these constraints primarily determine the broader operative framework between periareolar versus double-incision approaches and connected versus non-connected incisions. However, once patients are determined to be appropriate candidates for the double-incision approach, there is meaningful flexibility in scar design. Once stratified by non-connected and connected, surgeons can utilize any of the scar orientations assessed in this study as a viable option. Within this operative framework, the myriad scar orientations represent technically feasible variations and can ultimately be determined by patient preference.

While intuitively many patients may prefer a shape that follows a natural chest contour, aesthetic preference and gender expression are heterogeneous, and not all patients favor a curved or traditionally “natural-appearing” incision. Though our findings demonstrated a statistically significant preference for curved incisions, each incision type was ranked first at least once, highlighting meaningful variability in aesthetic goals. Notably, some patients may prefer more geometric or stylized scar patterns, including diagonal or inverse-diagonal configurations, which do not necessarily mimic native chest contours. Certainly, patient anatomy may be the arbiter of which incisions may look the most native- for example, in patients with a lean ectomorph body type, the angular incision would better approximate the contour of a defined pectoralis border than a gently curving incision. Nonetheless, the mere presence of preference variability seen in this study, even when a dominant pattern exists, underscores that patient perspectives should not be assumed based on aggregate trends. Instead, individual preferences should be explicitly elicited and incorporated into surgical planning. Indeed, although curved incisions are most commonly performed in the senior author’s practice, patients express preferences that differ from this convention, reinforcing the importance of highly individualized, tailored preoperative discussion.

In practice, these findings may be incorporated into preoperative consultation by providing patients with visual examples of different incision orientations to facilitate discussion of aesthetic goals and expectations. Among patients who have already been determined to be appropriate candidates for the double-incision approach, visual aids or preference sheets may be used to facilitate discussion regarding scar orientation preferences within the connected or non-connected configurations. Such discussions may help patients articulate aesthetic goals and identify which scar orientation most closely aligns with their desired chest appearance, while allowing the surgeon to discuss visual features (such as orientations that closely follow the pectoralis border, or others with more angular contours) and anticipated contribution to overall chest appearance. This approach to shared decision-making aligns with the broader movement toward patient-centered care in GAS and may help ensure that surgical planning more closely reflects individual patient preferences.^9^ Incorporation of patient preference is best viewed as a component of surgical planning within an established operative framework, rather than a determinant of operative candidacy. However, whether alignment between preferred and achieved scar orientation ultimately translates into improved postoperative outcomes remains unknown. Although preference data may provide valuable insight into patient perspectives regarding scar orientation, the present study did not evaluate postoperative satisfaction, gender congruence, quality of life, or other patient-reported outcomes. Specifically, this study was unable to assess whether concordance between a participant's preferred scar orientation and their actual postoperative scar pattern influences satisfaction with surgical outcomes. Future studies are needed to determine whether alignment between preferred and achieved scar orientation influences postoperative satisfaction and long-term perceptions of surgical outcomes.

Several limitations should be considered when interpreting these results. First, the predominance of patients who had already undergone surgery, particularly those who underwent double-incision mastectomy with free nipple grafting, introduces the possibility of confirmation bias, as respondents may preferentially rate scar patterns similar to their own outcomes. Next, because all participants were current or former patients of a single surgeon whose practice commonly employs curved incision patterns, familiarity with the surgeon’s typical aesthetic approach may have influenced aesthetic preferences, introducing the possibility of normalization bias. However, descriptive comparison of respondents with and without prior GAM demonstrated highly similar preference patterns, with identical rank ordering of non-connected incision designs and only minor differences among connected designs. Although these findings suggest that prior surgical experience alone may not fully explain the observed preference trends, surgeon-specific aesthetic practices remain a critical interpretive limitation. Because all participants were recruited from a single surgeon's practice, it is not possible to fully extricate patient preferences from prior exposure to a particular aesthetic approach. Multi-surgeon studies are warranted to determine whether similar preference patterns persist across diverse surgical practices and patient populations. More broadly, the relatively small sample size and single-surgeon cohort limit external validity, and findings derived from this selected population may reflect institutional or surgeon-specific practices that may not be generalizable to the broader transgender and gender-diverse population. The overall response rate was 23.5%, introducing the possibility of nonresponse bias, as individuals with stronger opinions regarding scar orientation may have been more likely to participate. Future multi-institutional studies incorporating a broader range of patient populations are needed to evaluate the consistency and generalizability of these findings.

Furthermore, while the use of schematic illustrations in the survey allowed for standardized comparison of scar orientation while minimizing potential confounding factors such as scar quality, pigmentation, contour irregularities, body habitus, nipple position, and postoperative healing characteristics, they do not fully replicate the appearance of real postoperative outcomes. As a result, participant preferences may differ when evaluating clinical photographs that incorporate these additional aesthetic variables. Subsequent studies incorporating representative postoperative photographs may provide complementary insight into scar orientation preferences when contextualized within real world surgical outcomes. Additionally, the cohort included a relatively limited number of non-binary participants. Although minor descriptive differences in incision preferences between transgender and non-binary respondents were observed, the small subgroup sample size precluded formal statistical analysis. These findings should be interpreted as exploratory and highlight the need for larger studies examining how diverse gender identities may shape surgical priorities. Finally, this study does not evaluate postoperative satisfaction, aesthetic outcomes, or revision rates, and therefore cannot determine whether preferred incision patterns are associated with improved clinical outcomes despite reaching statistical significance. As such, the relationship between stated aesthetic preferences and postoperative satisfaction remains unknown. Future prospective studies incorporating validated patient-reported outcome measures (PROMs), such as the TRANS-Q or other gender-affirming surgery–specific PROMs, are needed to assess whether alignment between preferred and actual incision design is associated with improved patient satisfaction and aesthetic outcomes.

Despite these limitations, this study highlights the importance of incorporating patient perspectives into surgical planning for double-incision GAM. In clinical practice, the senior author has encountered patients requesting several different scar orientations, suggesting that preferences for incision design are not uniform. Patients may also self-select surgeons whose typical incision style aligns with their personal aesthetic preferences. Increasing transparency and discussion of scar placement during preoperative consultation may therefore help align patient expectations with surgical outcomes.

## Conclusion

This study demonstrates that patients considering GAM have diverse preferences regarding scar orientation, with curved incisions following the pectoralis border emerging as the most consistently favored pattern. Incorporating discussion of scar orientation into preoperative counseling may support shared decision-making by helping align surgical planning with individual patient goals when multiple technically appropriate options exist within the constraints of patient anatomy.

## Ethical approval

This study was approved by the University of California, San Francisco Institutional Review Board (14–14,439) and conforms to the World Medical Association Declaration of Helsinki.

## Financial disclosure statement

None.

## Funding sources

None.

## Declaration of competing interest

None.
